# *Spirulina*-in Silico-Mutations and Their Comparative Analyses in the Metabolomics Scale by Using Proteome-Based Flux Balance Analysis

**DOI:** 10.3390/cells9092097

**Published:** 2020-09-15

**Authors:** Supatcha Lertampaiporn, Jittisak Senachak, Wassana Taenkaew, Chiraphan Khannapho, Apiradee Hongsthong

**Affiliations:** 1 Biochemical Engineering and Systems Biology Research Group, National Center for Genetic Engineering and Biotechnology, National Science and Technology Development Agency at King Mongkut’s University of Technology Thonburi, 49 Soi Thian Thale 25, Tha Kham, Bang Khun Thian, Bangkok 10150, Thailand; supatcha.ler@biotec.or.th (S.L.); jittisak.sen@biotec.or.th (J.S.); chiraphan.kha@biotec.or.th (C.K.); 2 Pilot Plant Development and Training Institute, King Mongkut’s University of Technology Thonburi, 49 Soi Thian Thale 25, Tha Kham, Bang Khun Thian, Bangkok 10150, Thailand; wassana.tae@kmutt.ac.th

**Keywords:** genome-scale, flux balance analysis, proteome analysis, temperature response, histidine kinase, in silico mutation

## Abstract

This study used an in silico metabolic engineering strategy for modifying the metabolic capabilities of *Spirulina* under specific conditions as an approach to modifying culture conditions in order to generate the intended outputs. In metabolic models, the basic metabolic fluxes in steady-state metabolic networks have generally been controlled by stoichiometric reactions; however, this approach does not consider the regulatory mechanism of the proteins responsible for the metabolic reactions. The protein regulatory network plays a critical role in the response to stresses, including environmental stress, encountered by an organism. Thus, the integration of the response mechanism of *Spirulina* to growth temperature stresses was investigated via simulation of a proteome-based GSMM, in which the boundaries were established by using protein expression levels obtained from quantitative proteomic analysis. The proteome-based flux balance analysis (FBA) under an optimal growth temperature (35 °C), a low growth temperature (22 °C) and a high growth temperature (40 °C) showed biomass yields that closely fit the experimental data obtained in previous research. Moreover, the response mechanism was analyzed by the integration of the proteome and protein–protein interaction (PPI) network, and those data were used to support in silico knockout/overexpression of selected proteins involved in the PPI network. The *Spirulina*, wild-type, proteome fluxes under different growth temperatures and those of mutants were compared, and the proteins/enzymes catalyzing the different flux levels were mapped onto their designated pathways for biological interpretation.

## 1. Introduction

Growth temperature stress causes biochemical changes in cells and reductions in biomass yield. Proteome analysis in *Spirulina* (*Arthrospira platensis*), a cyanobacterium, was performed to explore the changes at the protein level and the protein interaction cascade when the cells undergo many cellular modifications under thermal stress conditions. In previous proteome-wide studies, the identification of proteins regulated at both the translational and post-translational levels, that is, bi-level-regulated, could ideally represent the key signaling proteins that played a significant role in low- and high-temperature responses. The key two-component system (TCS) proteins SPLC1_S041070 (histidine kinase Hik28), SPLC1_S082010 and SPLC1_S230960 were identified as bi-level-regulated proteins and were linked to SPLC1_S270380 or glutamate synthase, an important enzyme in nitrogen assimilation that synthesizes glutamate from 2-oxoglutarate (2-OG), which is known as the signal compound that regulates the carbon/nitrogen (C/N) balance of cells. Therefore, the signaling mechanism can link to the metabolite 2-oxoglutarate, which controls the C/N balance and the key enzyme in the N assimilation of the cells [1,2]. Moreover, the integrated proteome- and protein–protein interaction data highlight the linkage of signaling proteins and proteins involved in nitrogen and ammonia assimilation, fatty acid desaturation, photosynthesis and oxidative stress.

Predictions of cell behavior under designated conditions can be achieved by integrating the metabolic modeling approach. Apart from fluxome analysis, metabolic models can be used as the backbone of the highly evolving field of integrative biology, extending from genome and transcriptome to proteome and metabolome. Flux balance analysis (FBA) is a mathematical technique that is used to simulate metabolism in the metabolic network of any organism using its genome-scale metabolic model. FBA is a “constraint-based” technique, of which metabolic fluxes in the steady-state metabolic network are controlled by stoichiometric reactions in the metabolic model. The possible answers obtained from the calculation can be reduced further by changing the values of maximum and minimum fluxes of each reaction, and the accuracy of possible answers can also be enhanced by adding more experimental fluxes into the calculation or by elucidating the answers from the calculation performed by arbitrarily entering the fluxes of some reactions into the simulation.

Regarding the metabolic model generated for *Spirulina,* first, Cogne et al. [3] proposed the small-scale metabolic model of *Spirulin*a with 121 reactions consisting of only the core metabolic pathways. Then, a model of 22 reactions focusing on lumped reactions in central metabolic pathways and GLA biosynthesis was reported [4]. In 2012, the first genome-scale metabolic model (GSMM) of *Spirulin*a was constructed by Klanchui et al. [5] containing 692 genes, 875 reactions and 753 metabolites and covering all the central metabolic pathways and biosynthetic pathways for some of the vitamins and cofactors. However, this GSMM treated all transport reactions as diffusion, and cellular compartments were divided only into the cytosol, periplasmic space and extracellular space. In the present study, the newly expanded GSMM of *Spirulina*, based on iAK692 [5], comprising 873 genes, 1444 reactions and 1151 metabolites was applied. The GSMM was improved by the incorporation of missing reactions and pathways, e.g., those in photosynthesis, tricarboxylic acid (TCA) cycle, transporters, prosthetic groups and coenzymes, as well as the deletion of the redundant ones. Moreover, in expanded GSMM, the major improvements are (i) compartmentalization of cellular compartments into six microcompartments [6] (ii) replacement of photosynthesis and oxidative phosphorylation pathways to include the photosynthetic linear electron flow (LEF) pathway [7], including photosystem II and I, alternate electron flow (AEF) pathways [8] and photorespiration [9], (iii) integration of novel TCA cycle reactions [10,11], (iv) inclusion of transport reactions and (v) expansion of biosynthetic pathways for vitamins and cofactors.

The application of proteomic data in genome-scale metabolic models is rarely employed, most likely due to limited access to high-throughput data and GSMM. Thus, a few reports on this particular integrated technique were carried out in model organisms, e.g., *Arabidopsis thaliana* and *Saccharomyces cerevisiae*, to investigate stress response mechanisms [12,13]. By using the protein level as constraints on a metabolic model, it should provide a more accurate/consistent snapshot of metabolism than the transcript level [14,15,16]. Recent publications have identified the stress-core proteome, which has contributed strongly to the accuracy of the prediction of combined temperature and light stress conditions [12]. Moreover, there is evidence that a large training set size with noise in the data may be detrimental to the flux predictions because the flux reaction bound may become too large due to data outliers [16]. The presence of promiscuous enzymes, isoenzymes, enzyme complexes, unknown gene associations and enzyme inactivation during regulatory interactions can also cause inconsistent and conflicting proteome levels in adjusting boundaries during gene-per-reaction mapping.

In the present study, we explored the use of genome-scale metabolic models (GSMM), constructed using proteomics data of significantly up- and downregulated proteins from *A. platensis* cultures grown under the stress conditions reported by our group [17,18,19], to understand the metabolic capabilities of the cells, through computational simulations and prediction of the response of the cyanobacteria to particular growth conditions. The proteome-based constraint method is applied to the newly expanded GSMM of *A. platensis* C1 to predict the flux distribution under three different temperature conditions to simulate the biological processes that occur under the temperature change. The model was constructed to simulate the genome-scale metabolic fluxes at the optimal temperature (35 °C) and under low- and high-temperature stress conditions (22 °C and 40 °C, respectively). The comparison of simulated metabolic flux under the three growth temperatures was performed to explore the reactions in metabolic pathways affected by the temperature changes. Moreover, the proof of concept was carried out by in silico knockout of the proteins involved in the temperature response mechanism regulated by the key signaling protein reported earlier, histidine kinase Hik28. The simulated fluxes of the knockout mutants were analyzed. The cascade of temperature stress response mechanisms at the metabolome-wide level was revealed. In the near future, the metabolic fluxes simulated from the proteome-based GSMM may lead to flux-oriented manipulation for maximum biomass production under environmental stress.

## 2. Materials and Methods

### 2.1. Proteome-Based Constraint Method

**Figure 1:**
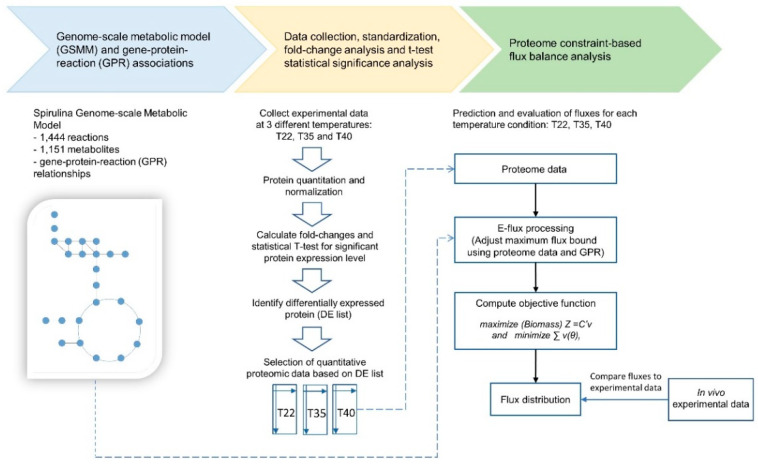
Workflow of the proteome-based metabolic model construction.

The protein expression level of *A. platensis* C1 was obtained from a proteomics analysis conducted with the three growth temperatures: an optimal temperature of 35 °C (T35), a low temperature of 22 °C (T22) and a high temperature of 40 °C (T40). After normalization of the quantitative proteome, the log2-fold changes in protein between the experimental temperature condition and control groups were computed. Then, a two-sample *t*-test was performed for statistical analysis. Proteins with a log-2-fold change greater than 1.2 and *p* < 0.05 were considered differentially expressed (DE) proteins [17,18,19]. Three DE protein lists were prepared from the comparisons between the stress temperatures, 22 °C and 40 °C, and the control temperature, 35 °C: the T22, T40 and T35 DE lists. The normalized expression level of the identified significantly DE proteins (according to DE protein list) was then used to determine the reaction flux constraints following the E-flux method. To determine the optimal flux distribution, linear programming was used to maximize the biomass, which was the objective function. A schematic of the proteome-based metabolic model construction is shown in Figure 1.

To incorporate the proteomic data into the metabolic flux analysis, we followed the process of the E-flux method, as described in Colijn et al. and Brandes et al. [20,21]. For each reaction, the reaction bounds were approximated proportionally to the proteomic level of the genes associated with each reaction. Proteomic data were mapped to corresponding reactions based on the gene–protein-reaction (GPR) association. When a protein has a measured expression level of value *p*, the allowable flux value through the respective reaction is set to [*–p*, *+p*] and [0, *+p*] for the reversible and irreversible reaction, respectively. When either the proteome or GPR for a certain reaction was unavailable, then the upper bounds were not constrained (a defined value was used in the model). If the reaction was catalyzed by multiple genes in an enzyme complex, the Boolean expression formula corresponding to enzyme complexes was utilized for that reaction. The mathematical operation for the Boolean operators “AND” and “OR” relationships were calculated as follows:

*X* “*AND*” *Y*: the minimum value of the two values; Min (X, Y);

*X* “*OR*” *Y*: the maximum value of the two values; Max (X, Y).

After constraining the metabolic model using the experimental proteomic data, the flux distribution of the model was predicted by solving the following linear optimization problem:

(1) maximize(Biomass)Z=C’v

(2) $$\mathrm{subject}\;\mathrm{to}\{\begin{array}{c} Sv=0\\ L_{i}\leq v_{i}\leq U_{i} \end{array}$$

where *Z*, the objective function, is the biomass production; *C’* is a row vector of the coefficient that defines the weight of each flux in the objective function; *S* is the stoichiometric matrix; *v* is a flux vector of n reactions, i.e., *v =* [*v_i_*; *I = 1, 2, 3 …, n*]; and *L_i_* and *U_i_* are the lower and upper flux bounds, respectively, for each reaction *i*, which is an approximation based on the normalized expression of the gene catalyzed in that reaction.

Since there were multiple feasible solutions within the constraints, in this case, the method found a unique metabolic flux distribution among the alternative optima by minimizing the total sum of absolute flux. Based on the assumption that the cell attempts to achieve its objective function while allocating the minimum amount of resources;

*minimize* ∑ |*v(θ)*_*i*_| (3)

where *v(θ)_i_* is a vector of reaction flux prediction given the proteome set *(θ).*

### 2.2. Metabolic Flux Simulation of the in Silico Knockout and Overexpression of Genes

According to the proteome-based genome-scale metabolic model (GSMM), the metabolic flux of *A. platensis* C1 under various in silico gene knockouts and overexpression was simulated. The gene knockout condition was simulated in the model by restricting the flux through the designated reactions by setting the upper and lower bounds of the corresponding reaction to zero. in silico overexpression was simulated by increasing or doubling the level of the upper and lower bounds of the corresponding reaction.

### 2.3. Comparative Analysis of the Metabolic Fluxes and Pathway Mapping

The present study is the first to present a comparative analysis of the metabolic fluxes obtained from proteome-based GSMM. Thus, the data normalization and the cut-off criteria for the significant change of the flux level have not been determined to date. In the case of flux values equaling zero, the original simulated values were used in the comparative analysis. Otherwise, the simulated fluxes obtained from the model were subjected to a log_2_ function. The log_2_-flux values were normalized with the standard deviation (SD) of each dataset. Second, the difference in the log_2_ of the simulated flux obtained from Conditions 1 and 2 was calculated as the fold change value. Third, the cut-off values for the significant change of the simulated metabolic fluxes compared between the two conditions were determined based on the proportion of the significant change reactions less than 30% of the total reaction number. Accordingly, in the case of temperature stresses and in silico knockout or overexpression, when the log_2_ values of simulated flux obtained from T35, T22 or T40 were used as the basal level, the cut-off fold change for the comparative analysis was 1.206.

Based on the KEGG global metabolic pathway as a template, the simulated metabolic fluxes of each condition were mapped on the designated reactions by using the KEGG Pathway Mapper tool. In the result images, other reactions that were not involved with the fluxes were manually eliminated.

## 3. Results

### 3.1. Comparative Analysis of the Simulated Proteome-based Fluxes and Their Correlation with Temperature Stress

**Figure 2:**
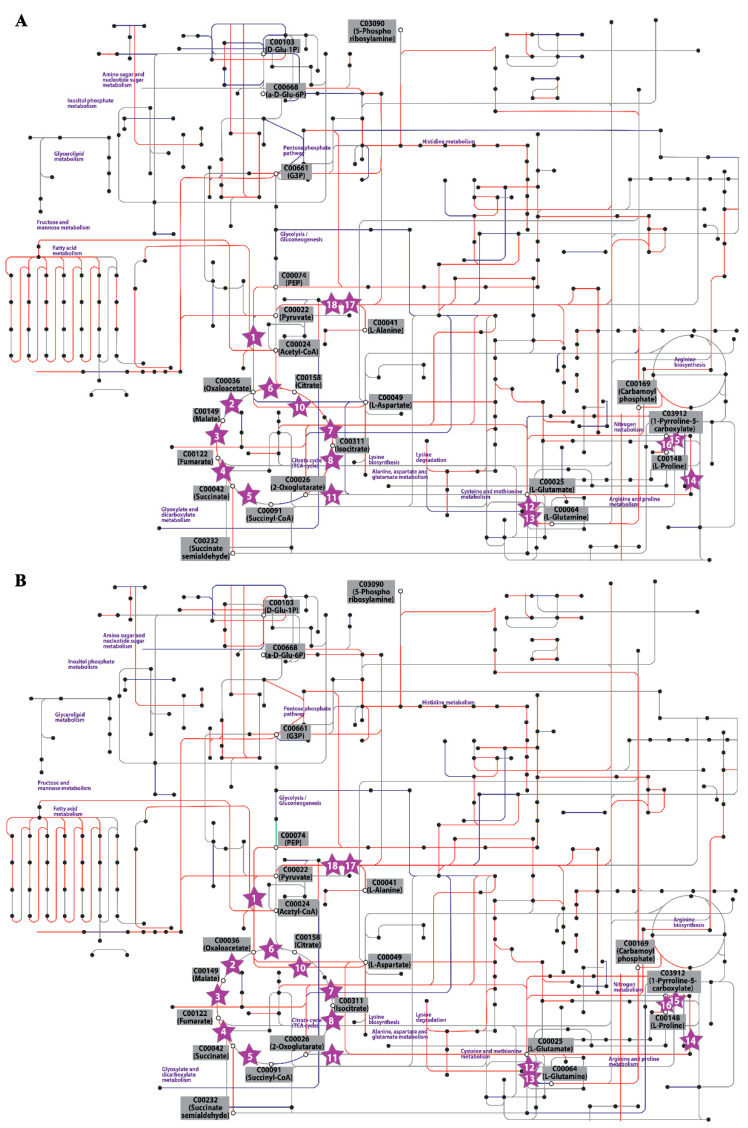
Central metabolic pathway mapping of the results obtained from the comparison of simulated metabolic flux when the cells were grown at (**A**) low temperature (22 °C) and (**B**) high temperature (40 °C), in comparison to that of the control (35 °C), on the template of KEGG -map01100, https://www.kegg.jp/kegg/pathway.html#global, by using the KEGG Mapper tool. The key enzymes (star-shaped nodes) are annotated with numbers, which are in the list of Figure 3. Major metabolites (black-bordered white circles) are labeled in gray boxes, while the pathway names are labeled in violet. The blue, red and light green lines represent upregulated, downregulated and unchanged or insignificantly changed fluxes. The gray line represents the reaction with a simulated flux level of ≤0.

The proteome-based constraint method is applied to the newly expanded GSMM as shown in Figure 1 and the metabolic fluxes simulated from proteome-based GSMM are shown in Table S1. The data clearly showed a decrease in the simulated biomass at 22 °C and 40 °C compared to that at the optimal temperature, 35 °C, which was well correlated with the experimental data obtained previously (Table S2). Regarding the overview of metabolic pathways, the simulated fluxes were mapped with the corresponding KEGG reactions, and the changes in global metabolism under the two stress temperatures are illustrated in Figure 2 and Figure S1.

To investigate the correlation of the temperature change and the simulated metabolic fluxes, scatter plots of the flux distribution were plotted between flux distributions at various temperature conditions. All the flux distribution profiles show a positive trend with a high correlation (Figure S2).

### 3.2. Simulated Flux Distribution Represented by Pathway Mapping

**Figure 3:**
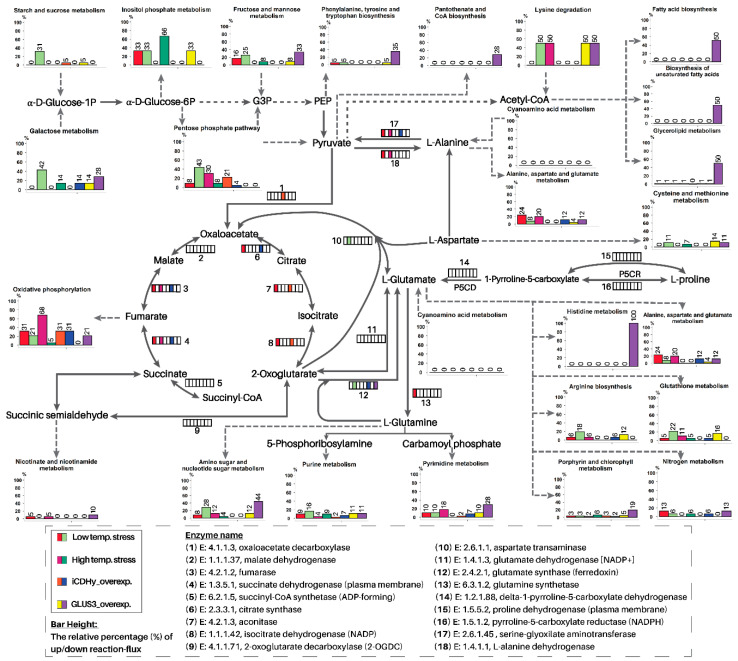
Schematic diagram representing the analysis of simulated metabolic flux of (i) the cells were grown at a low temperature (22 °C) and high temperature (40 °C), in comparison to that of the control, and (ii) the in silico overexpression of isocitrate dehydrogenase (iCDHy) and GlsF, in comparison to that of the control. The control is the simulated flux of the wild-type strain under optimal growth temperature at 35 °C. The central metabolic pathways, including those at the interconnection of carbon and nitrogen metabolism, were identified. The bar graphs illustrate the relative % of the affected reactions over the total reactions (100%) of each condition/mutant in the designated pathway. Note: Eight boxes in a row located over the reaction arrow represent the up and downregulated flux level compared between the treated and the control. The legends to up and downregulated flux (i) under the designated growth temperatures and (ii) of the in silico mutants are shown in the lower-left corner of the figure.

The simulated flux distribution was analyzed by pathway mapping using KEGG pathways as the templates (Figure S3). The affected flux levels that significantly changed after the temperature shift from 35 to 22 °C or from 35 to 40 °C were mapped onto the designated pathway. Then, the comparative results are summarized in Figure 3.

More than 90% of the flux level in some central metabolic pathways, e.g., glycolysis, the Calvin cycle and the pentose phosphate pathway, was decreased or unchanged when comparing the optimal- and high-temperature conditions. In contrast, an increasing flux level was observed in the central metabolic pathways after the low-temperature shift. However, the comparative analysis showed a decreasing level of fluxes in the TCA cycle, and as a result, 2-OG biosynthesis was possibly reduced under low-temperature stress. Moreover, the flux levels of the two glutamate (Glu)-synthesizing enzymes glutamate synthase and aspartate transaminase, one using 2-OG and the other using oxaloacetate as substrates, were upregulated at 22 °C.

### 3.3. In silico Knockout and Overexpression of Hik28 Client Proteins and the Proteins at the Interconnection of C- and N- Metabolism

The fluxes of the reactions catalyzed by three enzymes, isocitrate dehydrogenase (ICDHy), ferredoxin-dependent glutamate synthase (GLUS3) and glutamine synthetase (GLNS), which were found in the group of client proteins of Hik28 in the PPI network (Figure S4), were subjected to in silico knockout and overexpression. These three enzymes are directly involved with the 2-OG level. The changes in the overall simulated flux levels are presented in Table S3.

For example, an overview of metabolic flux analysis after the in silico knockout and overexpression of the reaction catalyzed by ICDHy, which presumably caused the reduction and enhancement of 2-OG, respectively, showed a decreasing flux of approximately 5% of the total metabolic reactions for both ICDHy mutants. However, approximately 4% of the flux level in the ICDHy-knockout and 7% of the ICDHy-overexpressing models was increased, and among these reactions, the flux catalyzed by GLUS3 was increased in the ICDHy-knockout model and vice versa in the ICDHy-overexpressing model. It is worth noting that the fluxes involved in CO_2_ uptake and oxygen evolution in photosystem II were induced in the ICDHy-overexpressing model, whereas an increase in citrate exchange flux was observed in both mutant models. Moreover, the exchange of 2-OG was also significantly enhanced as a result of the in silico overexpression of ICDHy. However, the biomass of these two mutant models was not changed.

In the case of overexpression of the reaction catalyzed by GLUS3 and GLNS, the simulated biomass was increased only in the GLUS3-overexpressing model. Similarly, the simulated fluxes involved in chlorophyll biosynthesis, photosystem II and fatty acid metabolism were induced after in silico knockout and overexpression (Table S3). It should be noted that the doubled level of GLUS3 flux has a positive effect on the majority of metabolic pathways, including cell biomass.

## 4. Discussion

### 4.1. Proteome-Based GSMM

To the best of our knowledge, this study is the first to employ experimental fold change proteomic datasets to represent relative change in *Spirulina* metabolism under an optimal growth temperature and suboptimal stress temperatures. The expression levels of significantly DE proteins were used as constraints for the new *Spirulina* metabolic flux model to provide an in silico-simulated snapshot of the metabolic changes that occur under different temperature conditions. Whether proteomics data are hard to obtain, and quantitative proteomic data is not widely available, the proteomics experiments are more precise and more suitable for metabolic integration than transcriptomics approaches. As reaction activities will correlate more strongly with protein abundance than with mRNA abundance, flux bound should be more correlated with protein expression than with mRNA expression. The estimated results from the integration of the proteomic dataset with the metabolic model enable more reliable metabolic flux predictions. Moreover, the estimated flux from the integration of a statistically significant differential expressed proteomic dataset into the metabolic model could refine our understanding of biological changes in *Spirulina* under temperature stress.

Accordingly, by using our proteome selection strategy, the flux predictions were in good agreement with experimental data obtained by our group and others, as described below. The simulated metabolic fluxes under the optimal temperature and the suboptimal stress temperatures obtained from the proteome-based GSMM correspond well to the experimental data obtained earlier. For example, the highest biomass of *A. platensis* C1 was observed under the optimal temperature compared to that under the stress conditions, and the biomass of the culture grown at 22 °C was higher than that at 40 °C (Table S2). Moreover, the fluxes in fatty acid desaturation under the stress temperatures confirmed the fatty acid composition obtained from the cultures under the designated growth temperatures [22,23].

### 4.2. Possible Biological Meaning Add Link Between Stories

Our previous proteome analysis of *A. platensis* C1 in response to temperature stress led to the hypothesis that the low-temperature stress represented a stress response pattern similar to that of nitrogen starvation due to changes in protein expression levels involved in nitrogen assimilation, e.g., GLUS3 [17,18,19]. According to the present proteome-based GSMM simulated using proteome data obtained from T22, the flux of GLUS3, aspartate transaminase (AspTA) and nitrite exchange involved in N assimilation were increased. Whereas the fluxes of the enzyme were reported to play a role under carbon limitation, ribulose-bisphosphate oxygenase and -carboxylase were reduced [1]. Moreover, under both temperature stresses, the simulated fluxes in chlorophyll biosynthesis and photosynthesis were reduced (Table S1), which corresponded well to the biomass reduction found in silico and in experiments. The data demonstrated that applying the proteome data obtained under stress to GSMM led to the reliable biological meaning of the simulated fluxes.

Proteome-based GSMM was then applied for in silico knockout and overexpression to mimic the biological conditions of interest. Under nitrogen starvation, the 2-OG level synthesized by a reversible enzyme, iCDHy, was reported to play a critical role as the signal molecule, which was reported to control the C/N balance, resulting in the N assimilation of the cells [24]. Thus, the increasing level of 2-OG was mimicked by in silico overexpression of the reaction that synthesizes the 2-OG performed by iCDHy, which presumably caused the accumulation of 2-OG (Table S3). The resulting fluxes of GLUS3 and ribulose-bisphosphate oxygenase and ribulose-bis carboxylase were upregulated under T22; however, the simulated fluxes of chlorophyll biosynthesis and photosynthesis were increased, and the simulated biomass was not affected by the in silico mutation. Moreover, it is well-known that under N depletion, arginine (Arg) biosynthesis is reduced, whereas fatty acid metabolism is induced [1]. The down- and upregulation of simulated fluxes in arginine biosynthesis and fatty acid metabolism, respectively, including desaturases, were detected only in the model of in silico overexpressed GLUS3, similar to that observed under nitrogen stress (Figure 3, Table S3). Li et al. reported that the control of C/N balance under N depletion occurred through the activity of N-acetyl-L-glutamate kinase (NACGK), which was regulated by the nitrogen regulatory protein PII. When the level of 2-OG was low, the PII interacted with NAGK and abolished the inhibitory effect of Arg on NACGK, thereby promoting its activity. In contrast, under N starvation, the increasing level of 2-OG, as a result of inhibited Arg biosynthesis, led to nitrogen assimilation and facilitated lipid biosynthesis [1].

After a comparative analysis of the T22, T40, iCDHy-overexpression and GLUS3-overexpression reactions, the results demonstrated that the reaction catalyzed by ribulose-bisphosphate carboxylase (RBPC), a key enzyme in carbon dioxide fixation, was the only downregulated reaction, whereas pyruvate kinases, PYK1, PYK5 and PYK6, and adenosylcobinamide kinase, ACBIKGTP, were the upregulated reactions found in common among the four models (Figure S5). Notably, the four upregulated metabolic fluxes, as mentioned above, were reported to be involved in the oxidative stress response [25,26].

Moreover, it was previously reported that the uncoupling of catabolism and anabolism could be an indicator of the delayed adaptation of bacterial cells to sudden unbalanced conditions [27,28]. In our proteome-based model, a reduction in biomass was found after temperature downshift and upshift, whereas a higher percentage of anabolic and catabolic reactions was downregulated than upregulated in the T40 model. This result showed the coupling of anabolism and catabolism and thus indicated the immediate adaptation of the cells to high-temperature stress. In the T22 model, the decrease in biomass was uncoupled, with a higher percentage of catabolic reactions being observed to be upregulated than downregulated; therefore, this finding might indicate the delayed adaptation of the cells to low-temperature stress. In the case of the in silico mutagenic model, biomass was increased in the model of GLUS3 overexpression in combination with anabolic and catabolic reactions. The results suggested immediate adaptation to the changing level of GLUS3 (Table S4).

An analysis of the ratio of the affected reactions in C- and N-metabolism after pathway mapping, as shown in Table S5, clearly shows that both temperature stresses caused greater upregulation of the reactions in nitrogen metabolism than that in carbon metabolism; thus, the C/N ratio was >1. The simulated data were well correlated with the findings of our previous quantitative proteome analysis [19]. It is worth noting that in silico overexpression of GLUS3 showed the highest number of upregulated reactions in both C- and N-metabolism. However, the highest number of downregulated reactions was observed in the in silico GLNS knockout. Moreover, the overexpression of iCDHy in the TCA cycle at the interconnection of C- and N-metabolism caused a more negative effect on the reaction fluxes in C-metabolism than in N-metabolism, resulting in a notably high C/N ratio.

### 4.3. Application to System Biology

The proteomic data obtained under growth temperature stress conditions were integrated to develop the proteome-based model in the present study. The model was subjected to biological applications in terms of gene manipulation and flux alteration in in silico knockout and overexpression.

In cyanobacteria, there have been intensive studies on 2-OG, which is the intermediate in the TCA cycle at the interconnection of C- and N-metabolism, as the signal molecule for nitrogen stress [24]. It is well-known that under nitrogen limitation, the level of 2-OG is increased and leads to the nitrogen assimilation of the cells via the control of nitrogen regulatory protein PII, and the reverse is observed under nitrogen oversupply. Thus, the C/N balance of the cells is maintained. GlsF and GlnA are enzymes that catalyze the following reactions of 2-OG biosynthesis. Moreover, these enzymes were found to be the client proteins of Hik28 in *A. platensis,* as mentioned earlier (Figure S4). Therefore, the proteome-based model was applied to simulate the fluxes in the cells where these two enzymes were missing or overexpressed.

The in silico knockout and overexpression of the reactions of interest can be performed by using proteome-based GSMM to simulate the systematic-scale fluxes that may have occurred after genetic manipulation. According to our previous proteomic analysis, several differentially expressed proteins under low-temperature stress were the same as those under nitrogen depletion, e.g., proteins involved in N assimilation and fatty acid desaturation [24]. Moreover, we found that Hik28, which played a role in the low-temperature stress response, showed a protein–protein association with several proteins involved in N assimilation, as shown in its PPI network (Figure S4). Protein binding experiments, yeast two-hybrid system, for Hik28 and its client proteins were performed by our group, and positive results were revealed [29]. Therefore, the proteome-based model was applied in this study to mimic the increasing level of the key signal molecule, 2-OG, under N-stress and the reaction fluxes that catalyzed the compounds surrounding the key reaction located at the interconnection of C- and N-metabolism. Metabolic changes associated with the in silico overexpression of iCDHy, GLUS3 and GLNS were analyzed as described earlier. The data obtained from the in silico mutant models was well corresponded to the effect of Hik28-deletion mutant to fatty acid analysis and photosynthetic activity under temperature stress, examined by our group (Kurdrid et al. [29] and Figures S6 and S7).

## 5. Conclusions

The GSMM is a computational analysis through FBA, which is used to predict and explain the performance and behavior of microorganisms. The mathematical approach is able to simulate metabolism providing information difficult to obtain in laboratory or field cultivations to maximize the production yield of microalgal biomass. In the present study, proteome analysis data obtained under growth temperature stress conditions of *A. platensis* C1 were integrated into our existing GSMM by using an optimization method. Taken together with the pathway mapping and analysis, the metabolic fluxes simulated from the proteome-based model led to prospective biological interpretation and enabled the prediction of genome-scale metabolic fluxes under the designated conditions, including those generated from in silico knockout and overexpression. Therefore, by using the model, the cultivated conditions for biomass production and production of the desired biochemical compounds, as well as the strategy for genetic manipulation for biochemical production, can be anticipated. However, the inability to directly integrate the cell regulatory mechanisms into the model remains a limitation regarding whether the proteome data obtained from stress response studies can be integrated into the GSMM. Moreover, the approach presented in this study is applicable to the in silico modification of a metabolic flux catalyzed by an irreversible enzyme.

## Supplementary Materials

The following are available online at https://www.mdpi.com/2073-4409/9/9/2097/s1, Figure S1: Global metabolic pathway mapping of the results obtained from the comparison of simulated metabolic flux when the cells were grown at (A) low temperature (22 °C) and (B) high temperature (40 °C), Figure S2: Scattering plot representing the correlation of the growth temperature and the simulated flux level, Figure S3: Bar graph illustrating the relative % of the affected reaction fluxes compared to the total reactions (100%), simulated at 22 °C and 40 °C in the designated pathway map, Figure S4: Protein-protein interaction network of Hik28 constructed by using STRING, Figure S5: Venn diagram showing the number of the affected reactions under/of the simulated conditions/in silico mutants, Figure S6: Graph of the fatty acid composition and GC profiles of total fatty acids, Figure S7: The amounts of chlorophyll a and photosynthetic oxygen-evolving activity in wild type and mutant, Table S1: Metabolic reactions and their simulated flux of the *Spirulina*-GSMM under the optimal growth temperature of 35 °C (T35), low temperature of 22 °C (T22) and high temperature of 40 °C (T40), Table S2: The effect of temperature on specific growth rate and biomass concentration of *Arthrospira platensis* strains, Table S3: Metabolic reactions of *Spirulina*-GSMM and their simulated flux of the in silico knockout/overexpress mutants, Table S4: Classification of reactions into catabolism and anabolism, Table S5: Ratio of the relative % of affected reaction in the carbon and nitrogen metabolism under/of each condition/in silico mutant.

## References

[1] Li Y., Liu W., Sun L.-P., Zhou Z.-G. (2017). Evidence for PII with NAGK interaction that regulates Arg synthesis in the microalga *Myrmecia incisa* in response to nitrogen starvation. Sci. Rep..

[2] Domínguez-Martín M.A., López-Lozano A., Clavería-Gimeno R., Velázquez-Campoy A., Seidel G., Burkovski A., Díez J., García-Fernández J.M. (2018). Differential NtcA responsiveness to 2-oxoglutarate underlies the diversity of C/N balance regulation in *Prochlorococcus*. Front. Microbiol..

[3] Cogne G., Gros J.B., Dussap C.G. (2003). Identification of a metabolic network structure representative of *Arthrospira (Spirulina) platensis* metabolism. Biotechnol. Bioeng..

[4] Meechai A., Pongakarakun S., Deshnium P., Cheevadhanarak S., Bhumiratana S. (2004). Metabolic flux distribution for γ-linolenic acid synthetic pathways in *Spirulina platensis*. Biotechnol. Bioprocess Eng..

[5] Klanchui A., Khannapho C., Phodee A., Cheevadhanarak S., Meechai A. (2012). iAK692: A genome-scale metabolic model of *Spirulina platensis* C1. BMC Syst. Biol..

[6] Vonshak A., Vonshak A. (1997). Spirulina Platensis (Arthrospira): Physiology, Cell-Biology and Biotechnology.

[7] Kramer D.M., Evans J.R. (2011). The importance of energy balance in improving photosynthetic productivity. Plant Physiol..

[8] Nogales J., Gudmundsson S., Knight E.M., Palsson B.O., Thiele I. (2012). Detailing the optimality of photosynthesis in cyanobacteria through systems biology analysis. Proc. Natl. Acad. Sci. USA.

[9] Cooley J.W., Vermaas W.F. (2001). Succinate dehydrogenase and other respiratory pathways in thylakoid membranes of *Synechocystis* sp. strain PCC 6803: Capacity comparisons and physiological function. J. Bacteriol..

[10] Steinhauser D., Fernie A.R., Araujo W.L. (2012). Unusual cyanobacterial TCA cycles: Not broken just different. Trends Plant Sci..

[11] Zhang S., Bryant D.A. (2011). The tricarboxylic acid cycle in cyanobacteria. Science.

[12] Fürtauer L., Pschenitschnigg A., Scharkosi H., Weckwerth W., Nägele T. (2018). Combined multivariate analysis and machine learning reveals a predictive module of metabolic stress response in *Arabidopsis thaliana*. Mol. Omics.

[13] Zelezniak A., Vowinckel J., Capuano F., Messner C.B., Demichev V., Polowsky N., Mulleder M., Kamrad S., Klaus B., Keller M.A. (2018). Machine learning predicts the yeast metabolome from the quantitative proteome of kinase knockouts. Cell Syst..

[14] Montezano D., Meek L., Gupta R., Bermudez L.E., Bermudez J.C.M. (2015). Flux balance analysis with objective function defined by proteomics data-metabolism of *Mycobacterium tuberculosis* exposed to mefloquine. PLoS ONE.

[15] Großeholz R., Koh C.C., Veith N., Fiedler T., Strauss M., Olivier B., Collins B., Schubert O., Bergmann F., Kreikemeyer B. (2016). Integrating highly quantitative proteomics and genome-scale metabolic modeling to study pH adaptation in the human pathogen *Enterococcus faecalis*. NPJ Syst. Biol. Appl..

[16] Tian M., Reed J.L. (2018). Integrating proteomic or transcriptomic data into metabolic models using linear bound flux balance analysis. Bioinformatics.

[17] Hongsthong A., Sirijuntarut M., Prommeenate P., Lertladaluck K., Porkaew K., Cheevadhanarak S., Tanticharoen M. (2008). Proteome analysis at the subcellular level of the cyanobacterium *Spirulina platensis* in response to low-temperature stress conditions. FEMS Microbiol. Lett..

[18] Hongsthong A., Sirijuntarut M., Yutthanasirikul R., Senachak J., Kurdrid P., Cheevadhanarak S., Tanticharoen M. (2009). Subcellular proteomic characterization of the high-temperature stress response of the cyanobacterium *Spirulina platensis*. Proteome Sci..

[19] Kurdrid P., Senachak J., Sirijuntarut M., Yutthanasirikul R., Phuengcharoen P., Jeamton W., Roytrakul S., Cheevadhanarak S., Hongsthong A. (2011). Comparative analysis of the *Spirulina platensis* subcellular proteome in response to low- and high-temperature stresses: Uncovering cross-talk of signaling components. Proteome Sci..

[20] Colijn C., Brandes A., Zucker J., Lun D.S., Weiner B., Farhat M.R., Cheng T.Y., Moody D.B., Murray M., Galagan J.E. (2009). Interpreting expression data with metabolic flux models: Predicting *Mycobacterium tuberculosis* mycolic acid production. PLoS Comput. Biol..

[21] Brandes A., Lun D.S., Ip K., Zucker J., Colijn C., Weiner B., Galagan J.E. (2012). Inferring carbon sources from gene expression profiles using metabolic flux models. PLoS ONE.

[22] Kurdrid P., Subudhi S., Hongsthong A., Ruengjitchatchawalya M., Tanticharoen M. (2005). Functional expression of *Spirulina*-Delta6 desaturase gene in yeast, *Saccharomyces cerevisiae*. Mol. Biol. Rep..

[23] Hongsthong A., Deshnium P., Paithoonrangsarid K., Cheevadhanarak S., Tanticharoen M. (2003). Differential responses of three acyl-lipid desaturases to immediate temperature reduction occurring in two lipid membranes of *Spirulina platensis* strain C1. J. Biosci. Bioeng..

[24] Oliveira M.A., Gerhardt E.C., Huergo L.F., Souza E.M., Pedrosa F.O., Chubatsu L.S. (2015). 2-Oxoglutarate levels control adenosine nucleotide binding by *Herbaspirillum seropedicae* PII proteins. FEBS J..

[25] Ferrer A., Rivera J., Zapata C., Norambuena J., Sandoval A., Chávez R., Orellana O., Levicán G. (2016). Cobalamin Protection against Oxidative Stress in the Acidophilic Iron-oxidizing Bacterium *Leptospirillum* Group II CF-1. Front. Microbiol..

[26] Ma X.C., Zhu S.Y., Luo M.M., Hu X.C., Peng C., Huang H., Ren L.J. (2019). Intracellular response of *Bacillus natto* in response to different oxygen supply and its influence on menaquinone-7 biosynthesis. Bioprocess Biosyst. Eng..

[27] Weber J., Hoffmann F., Rinas U. (2002). Metabolic adaptation of *Escherichia coli* during temperature-induced recombinant protein production: 2. Redirection of metabolic fluxes. Biotechnol. Bioeng..

[28] Weber J., Kayser A., Rinas U. (2005). Metabolic flux analysis of *Escherichia coli* in glucose-limited continuous culture. II. Dynamic response to famine and feast, activation of the methylglyoxal pathway and oscillatory behaviour. Microbiology.

[29] Kurdrid P., Phuengcharoen P., Senachak J., Saree S., Hongsthong A. (2020). Revealing the key point of the temperature stress response of *Arthrospira platensis* C1 at the interconnection of C- and N- metabolism by proteome analyses and PPI networking. BMC Mol. Cell Biol..

